# *RBDtector*: an open-source software to detect REM sleep without atonia according to visual scoring criteria

**DOI:** 10.1038/s41598-022-25163-9

**Published:** 2022-12-03

**Authors:** Annika Röthenbacher, Matteo Cesari, Christopher E. J. Doppler, Niels Okkels, Nele Willemsen, Nora Sembowski, Aline Seger, Marie Lindner, Corinna Brune, Ambra Stefani, Birgit Högl, Stephan Bialonski, Per Borghammer, Gereon R. Fink, Martin Schober, Michael Sommerauer

**Affiliations:** 1 Institute of Neuroscience and Medicine (INM-1), Forschungszentrum Jülich, Jülich, Germany; 2 Department of Neurology, Medical University of Innsbruck, Innsbruck, Austria; 3 Department of Neurology, Faculty of Medicine, University Hospital Cologne, University of Cologne, Cologne, Germany; 4 Institute of Neuroscience and Medicine (INM-3), Forschungszentrum Jülich, Leo-Brandt-Str. 5, 52425 Jülich, Germany; 5 Department of Nuclear Medicine and PET Centre, Aarhus University Hospital, Aarhus, Denmark; 6 Department of Neurology, Aarhus University Hospital, Aarhus, Denmark; 7 Department of Clinical Medicine, Aarhus University, Aarhus, Denmark; 8 Department of Medical Engineering and Technomathematics, FH Aachen University of Applied Sciences, Jülich, Germany; 9 Institute for Data-Driven Technologies, FH Aachen University of Applied Sciences, Jülich, Germany

**Keywords:** Sleep disorders, Parkinson's disease, Parkinson's disease, Software

## Abstract

REM sleep without atonia (RSWA) is a key feature for the diagnosis of rapid eye movement (REM) sleep behaviour disorder (RBD). We introduce *RBDtector*, a novel open-source software to score RSWA according to established SINBAR visual scoring criteria. We assessed muscle activity of the mentalis, flexor digitorum superficialis (FDS), and anterior tibialis (AT) muscles. RSWA was scored manually as tonic, phasic, and any activity by human scorers as well as using *RBDtector* in 20 subjects. Subsequently, 174 subjects (72 without RBD and 102 with RBD) were analysed with *RBDtector* to show the algorithm’s applicability. We additionally compared *RBDtector* estimates to a previously published dataset. *RBDtector* showed robust conformity with human scorings. The highest congruency was achieved for phasic and any activity of the FDS. Combining mentalis any and FDS any, *RBDtector* identified RBD subjects with 100% specificity and 96% sensitivity applying a cut-off of 20.6%. Comparable performance was obtained without manual artefact removal. RBD subjects also showed muscle bouts of higher amplitude and longer duration. *RBDtector* provides estimates of tonic, phasic, and any activity comparable to human scorings. *RBDtector*, which is freely available*,* can help identify RBD subjects and provides reliable RSWA metrics.

## Introduction

The hallmark of Rapid Eye Movement (REM) sleep behaviour disorder (RBD) is a failure to efficiently suppress motor activity during REM sleep, resulting in REM sleep without atonia (RSWA) and eventually the acting out of dream content^1,2^. RBD is strongly associated with α-synucleinopathies, namely Parkinson’s disease (PD), dementia with Lewy bodies (DLB) and multiple system atrophy (MSA), but can also occur in other neurological disorders^3,4^. Patients with isolated RBD (= iRBD patients) are deemed to be in a very early stage of an α-synucleinopathy and are at high risk to phenoconvert to PD, DLB, or, rarely, MSA^5^. This opens a unique window to study the processes during the early stages of these neurodegenerative diseases^6,7^. Additionally, PD patients with RBD often show a more aggressive phenotype, reflected by faster motor progression, and a higher probability of cognitive impairment as well as autonomic disturbances^8,9^. If RBD is present at PD diagnosis, this might even indicate a specific subtype of α-synuclein spread^10–14^. Extending the approach of subtyping PD patients according to their RBD status, a growing number of reports have stressed the significance of RSWA quantification as a severity marker^5,10,15,16^. Hence, correct diagnosis of RBD and reliable quantification of RSWA are paramount in the context of early PD and α-synucleinopathies in general^17^.

In 2012, the SINBAR (Sleep Innsbruck Barcelona) group published normative RSWA values for the diagnosis of RBD based on a study of 30 RBD patients and 30 matched controls. Their results have been incorporated into the current American Academy of Sleep Medicine (AASM) manual for scoring of sleep and associated events and into the video-polysomnography guidelines for RBD diagnosis of the International RBD Study Group^17–19^. The SINBAR criteria constituted the first and fundamental approach to include electromyography (EMG) from multiple muscles. They particularly propose that EMG activity of the arms is specific for RBD, and better suited to identify RBD than chin EMG activity alone^17,18,20,21^. However, visual assessments of RSWA are highly time-consuming and ratings vary considerably across scorers^22^. Therefore, computer-based automatized RSWA scoring methods have been evaluated^23,24^. Unfortunately, these methods only show limited inter-method agreement, often require costly commercial software, and nearly exclusively focus on chin EMG activity, which is particularly vulnerable to artefacts^20^.

Following recent International RBD Study Group guidelines^17^, we aimed at developing a software algorithm that (i) implements a well-established visual scoring scheme to detect and quantify RSWA, (ii) allows for the analysis of EMG from multiple muscles including the chin, arm, and leg muscles, and (iii) provides an open-source tool in a free software package reading PSG data in the European Data Format (EDF) for best applicableness.

We compared the performance of *RBDtector* with visual scorings from a sleep expert and a doctoral candidate in sleep medicine (“novice scorer”) and provide RSWA values from a large dataset of controls, patients with iRBD, and PD patients with and without RBD (n = 174). We evaluated the effects of common artefacts on the different EMG channels and investigated additional RSWA metrics, such as amplitude and duration of muscle bouts. Finally, we compared *RBDtector* estimates to a previously published dataset of 80 subjects from the SINBAR group^25^.

## Methods

### Participants and polysomnography recordings

All data were acquired from two case–control studies including control subjects, PD patients and iRBD patients who underwent overnight-video-polysomnography (PSG). All iRBD patients were recruited from the general population through newspaper advertisements. Following a structured telephone interview, subjects with a high likelihood of having RBD were invited to undergo PSG. Subjects in whom iRBD was excluded by video-PSG were considered control subjects in the further analysis. PD patients were recruited via an advertisement in the German Parkinson’s disease magazine, from local self-help groups, and the outpatient clinic of the University Hospital Cologne. All PSGs were evaluated by a board-certified sleep expert (MS). Inclusion criteria comprised the following: age 40–80 years (PD patients 50–80 years), no diagnosis of dementia, no severe comorbidity (e.g. cancer, end-stage renal or liver disease), no known stroke or traumatic brain injury. Medication was assessed and hypnotics, antidepressants or neuroleptics had to be discontinued two weeks before PSG in all non-PD subjects. PD was diagnosed according to the current Movement Disorders Society guidelines^26^. Both studies were approved by the local ethical committee (Ethics Commission of Cologne University’s Faculty of Medicine). All participants gave written, informed consent before participating. The work was carried out under the Declaration of Helsinki.

All PSGs were undertaken at home or in a hotel room. We used a mobile SOMNOscreen™ plus device for overnight video-PSG including ten EEG recordings (according to the international 10/20 system: F3, F4, C3, C4, O1, O2, A1, A2, Fpz as grounding, and Cz as reference), electrooculography, surface EMG of the mentalis, the tibialis anterior (TA), and flexor digitorum superficialis muscles (FDS, acquired in n = 143 (= 85.1% of all) PSGs), electrocardiography, nasal pressure and flow monitoring, thoracic and abdominal respiratory effort belts, finger pulse oximetry, and synchronized audio-visual recording. Before turning the lights off, electrode impedances were checked to be lower than 10kΩ. All EMG channels were recorded at a sampling rate of 256 Hz. 10 Hz high pass, 100 Hz lowpass filters, and a 50 Hz notch filter were applied before analysis.

Visual PSG scoring was performed on 30-s epochs including sleep efficiency, total sleep time, the absolute amounts of stage 1 (N1), stage 2 (N2), stage 3 (N3), and REM sleep, the apnea–hypopnea index (AHI, number of apnea plus hypopnea events per hour of sleep), and the periodic limb movement index (PLMI, number of periodic leg movements per hour of sleep) according to the AASM Manual for the Scoring of Sleep and Associated Events Version 2.6^19^. Diagnosis of RBD was made according to the International Classification of Sleep Disorders (ICSD)-3 criteria^27^. Visual PSG scoring and diagnosis of RBD was done by MS, who is a board-certified sleep expert. The evaluation was blinded from the visual RSWA scorings of NO and NW as well as from the *RBDtector* results. RBD diagnosis of the sleep expert was used as the gold standard for assessments of classification performance. Final diagnosis could not be achieved in five subjects due to technical failure of video-recording (n = 1), increased RSWA without anamnestic or video-recorded enactment of dream content (n = 1), or severe REM-associated sleep apnea making it impossible to judge muscle activity (n = 3). These PSGs were discarded from further analysis.

### Visual RSWA scoring

Rules for RSWA scoring were adopted from the original publication of the SINBAR group from 2012 and applied to all EMG channels in the same way^18^: Increased muscle activity was defined as EMG activity with an amplitude of at least twice the background muscle tone (= baseline) for at least 100 ms. An interval of more than 250 ms with baseline activity defined the end of increased EMG activity. Depending on the duration of increased muscle activity, we scored ‘phasic’ bouts for activity lasting between 0.1 and 5.0 s and ‘intermediate’ bouts for activity lasting between 5.1 and 15 s (needed to calculate any activity). Each 30 s sleep epoch was subdivided into ten 3 s mini-epochs and each mini-epoch was counted as phasic if it contained at least one ‘phasic’ bout. Each mini-epoch was scored as comprising any activity if any ‘phasic’, ‘intermediate’, or ‘tonic’ bouts were present. We scored a 30 s epoch with tonic activity if increased EMG activity was present in more than 50% of the total 30 s epoch—this could be achieved by a sustained increase of EMG activity for more than 15 s without an interruption of 250 ms of baseline activity (= ‘tonic’ bout) and by ‘phasic’ and ‘intermediate’ bouts exceeding 15 s during a 30 s epoch. To identify a ‘phasic’ or ‘intermediate’ bout superimposing a sustained tonic activity, it was required that the ‘phasic’ or ‘intermediate’ bout had at least twice the amplitude of the background tonic activity.

Visual scoring was performed by a sleep expert (NO, “expert scorer”) with multiple years of experience in clinical sleep medicine and by a doctoral candidate in sleep medicine (NW, “novice scorer”), who was introduced to the SINBAR scoring system and scored EMGs of 20 PSGs supervised by MS. We included two scorers with different levels of training to acknowledge that visual RSWA scoring is not only scorer-dependent but might also be dependent on the expertise of an individual scorer^22^. To ensure a common understanding of the rating system, both scorers were allowed to rate 5 PSGs with, and 5 PSGs without RBD, unblinded to RBD-status and to the scorings of the other scorer. Ratings were jointly discussed with MS as an independent referee to agree on judgements between scorers.

We randomly selected 10 iRBD patients and 10 controls for blinded visual RSWA scoring on mentalis, FDS, and TA EMG for comparison with the algorithm’s results. Additionally, both human scorers rated EMG activity as artefacts, if they did not consider the activity as caused by physiological muscle activity (examples of artefacts include snoring, technical issues, respiration, and electrocardiography). The combined artefact intervals of both human scorers were excluded from the inter-rater comparisons. Arousals had been identified during routine PSG scoring and were excluded. Ratings were executed using DOMINO software (SOMNOmedics, Randersacker, Germany) and exported for further analysis.

### Implementation of RSWA scoring to a computer algorithm

*RBDtector* is based on Python 3.8 (including Python libraries pandas and PyEDFlib) and reads EMG data in European Data Format (EDF). The sleep profile, arousal events, and optionally respiratory events as well as snoring artefacts are gathered from plain text files. For this study specifically, data were exported from DOMINO software.

EMG data are resampled to 256 Hz using spline interpolation if not already recorded at this sampling rate. REM sleep periods are extracted from the sleep profile. Previously identified arousals and respiratory events are handled as artefacts and discarded in all EMG channels, whereas periods of snoring are only considered for (and excluded in) mentalis EMG.

Subsequently, automated baseline detection of each ‘REM sleep period’ is performed separately. By definition, a ‘REM sleep period’ must contain continuous REM sleep for ≥ 150 s. REM sleep with EMG activity with a root mean square (RMS) < 0.05 mV is handled as an artefact (i.e. electrode detachment) and excluded from further analysis. Next, each ‘REM sleep period’ is assigned the amplitude with the lowest RMS of a 30 s rolling window from a continuously artefact-free (without arousal and respiratory events as well as snoring) REM period as ‘baseline amplitude’. Periods of REM sleep that do not meet these criteria are assigned to the ‘baseline amplitude’ of the previous REM period or the following if no previous one is available. If no ‘baseline amplitude’ can be estimated, the process is repeated with a rolling window size of 15 s. If this process is unsuccessful, the affected channel is discarded from further analysis. REM bouts shorter than 150 s are assigned the ‘baseline amplitude’ of the nearest REM period.

After excluding artefacts and determining ‘baseline amplitudes’, the SINBAR RSWA scoring rules are applied to all EMG channels stepwise. First, amplitudes for individual 30 ms bouts are defined. For that purpose, *RBDtector* calculates the RMS of the EMG amplitudes within a sliding window of 30 ms length, beginning at an artefact-free REM bout. The sliding window is shifted with a step size of 15 ms, yielding RMS values at a temporal resolution of 15 ms. 30 ms REM bouts with amplitudes exceeding two times the ‘baseline amplitude’ are considered ‘activity bouts’. Next, connected ‘activity bouts’ lasting longer than 0.1 s are identified and considered as an ‘activity event’. To those ‘activity events’, preceding and following ‘activity bouts’ are added, if no interruption occurs that has less activity than twice the ‘baseline amplitude’ for ≥ 0.25 s. This process is repeated until interruptions of ≥ 0.25 s with activity less than twice the ‘baseline amplitude’ are eventually identified (= ‘increased activity’).

In the next step, RSWA is classified as tonic, phasic or any. For scoring tonic activity, each 30 s epoch of REM sleep is examined for containing ≥ 50% of ‘increased activity’. If tonic activity is scored in one epoch, the baseline value of this 30 s epoch is elevated to the RMS of the period of tonic activity. Subsequently, if tonic activity is identified, ‘increased activity’ to identify phasic activity is recalculated in this channel to account for the changes in the baseline.

Phasic activity is detected if ‘increased activity’ persists ≤ 5 s. Phasic events are calculated by subdividing 30 s macro epochs into ten 3 s mini epochs and each mini epoch containing ‘increased activity’ is scored as phasic activity. Mini epochs with any activity are detected by combining all 30 s macro epochs with tonic activity and all 3 s mini epochs containing ‘increased activity’ between 0.1 and 15 s.

For phasic and any activity, the maximum amplitude and duration of “increased activity” bouts are determined and the respective arithmetic mean of all bouts per channel is computed.

Finally, a CSV file with the exact event timestamps and two xlsx files containing the SINBAR event evaluation data are created. The first xlsx file includes phasic, tonic and any (mini-) epochs per evaluated EMG channel, both in absolute numbers and in percent of the channel’s artefact-free REM sleep (mini-)epochs. Additionally, the mean values of maximum amplitude and duration for the phasic and any events are given, and the total amounts of REM sleep (mini-)epochs with and without the relevant artefacts. The second xlsx file depicts the percentage of RSWA events on combined channels relative to the amount of artefact-free REM sleep.

*RBDtector* is available at: https://github.com/aroethen/RBDtector. A compressed file of *RBDtector* is also part of the supplement.

### Statistical analysis

We explored the data with Statistical Package for the Social Sciences (SPSS) version 28. Group data are presented as mean ± standard deviation or relative frequencies unless otherwise stated. Normal data distribution was assessed with the Shapiro–Wilk test, Q-Q plots, and box plots. Group comparisons were calculated using Student’s *t*, Mann–Whitney *U*, and chi-square tests as well as analyses of variances and Kruskal–Wallis tests as appropriate. Univariate correlation analyses were calculated using Spearman’s *rho*. Coefficients of determination (R^2^) were used to compare inter-rater variability on a subject level (= amount of RSWA as a percentage of total REM sleep of the given subject) and Cohen’s kappa on a single epoch level (= binary single 3 s mini-, and 30 s epoch scorings, respectively). We analysed discrimination performance with receiver operating characteristic (ROC) curves. Areas under the curve (AUC) were calculated for each analysis and cut-off thresholds are given for the highest specificity. Additionally, we calculated sensitivity and accuracy at that threshold. Significance was accepted at *p* < 0.05.

We also compared *RBDtector* RSWA estimates on data from a previous study of the SINBAR group^25^. Inclusion and exclusion criteria, acquisition of PSG data and analysis as well as methods for RSWA quantification are described elsewhere^25^.

## Results

### Comparison of human scorings and RBDtector results

Clinical and demographic data as well as PSG characteristics of the arbitrarily selected 10 RBD-positive patients and 10 controls for inter-scorer and *RBDtector* comparison are reported in Table 1.

**Table 1:** Demographics and polysomnography data of 10 + 10 subjects for comparing human scorings.

	RBD n = 10	controls n = 10	p-value
**Demographics**			
Age [y]	69.0 ± 5.7	60.0 ± 12.3	0.061*
Sex [m/f]	7/3	9/1	0.582^#^
BMI [kg/m^2^]	25.6 ± 2.1	25.3 ± 2.8	0.796^§^
**Polysomnography**			
Total bedtime [min]	439.4 ± 67.7	422.0 ± 90.2	0.649*
Sleep efficiency [%]	85.4 ± 9.6	86.1 ± 8.3	0.912^§^
Sleep latency (N2) [min]	14.6 ± 16.4	10.5 ± 5.2	1.000^§^
N1 [min]	90.2 ± 65.8	71.0 ± 43.8	0.796^§^
N2 [min]	160.5 ± 36.6	184.6 ± 54.6	0.278*
SWS [min]	57.9 ± 26.4	46.4 ± 24.3	0.336*
REM [min]	56.8 ± 25.8	57.8 ± 25.1	0.931*
AHI [/h]	6.2 ± 8.0	10.6 ± 13.3	0.393^§^
PLMSI [/h]	43.7 ± 32.2	29.7 ± 34.7	0.315^§^

*BMI* body mass index, *N1/2* non-rapid eye movement sleep stage 1/2, *SWS* slow wave sleep, *REM* rapid eye movement sleep, *RBD* REM sleep behaviour disorder, *AHI* apnoea hypopnea index, *PLMSI* periodic limb movements in sleep index.

Statistics: *Student’s *t* test, ^§^Mann–Whitney U test, ^#^Chi-square test.

After the elimination of arousal events, 1769 30 s epochs (= 74.7% of all REM epochs) and 21,289 3 s mini-epochs (= 89.9% of all REM mini-epochs) were evaluated for tonic, phasic, and any activity as well as for non-physiological artefacts in the chin, FDS, and TA EMG by two human scorers. Most artefacts were identified in the mentalis channel (24.2 ± 28.9% of 3 s mini-epochs, range 0–94.0%; H(4) = 29.636, p < 0.001), whereas artefacts in the arm and leg electrodes were infrequent (average of all channels: 1.2 ± 1.2%, range 0 – 6.8%). Agreement between scorers on artefacts was poor, and κ values ranged between 0.21 – 0.30 across all channels.

After the elimination of the combined artefacts from both scorers, the amount of RSWA in patients and controls did not differ between the scorings of the human expert and *RBDtector* across all channels at group level. However, we observed lower estimates of RSWA of the novice scorer compared to *RBDtector* for any activity at the mentalis, right FDS, and right TA as well as for right TA phasic activity. Human scorings did not differ significantly across scorers, but again estimates of RSWA based on the analysis of the novice scorer were numerically lower (Table 2). On average, the ten RBD-positive patients showed elevated muscle activity in the mentalis and FDS, whereas TA EMG scorings showed only minor differences between groups (Table 2).

**Table 2:** RSWA indices of RBD-positive patients and controls evaluated by human scorers and the RBDtector.

	RBDtector		Expert scorer		Novice scorer	
	RBD	controls	RBD	controls	RBD	controls
**Mentalis**						
Tonic [%]	11.5 ± 16.1*	0.0 ± 0.0	11.7 ± 16.3*	0.0 ± 0.0	2.7 ± 4.2	0.2 ± 0.6
Phasic [%]	19.9 ± 9.7*	4.6 ± 3.4	20.6 ± 9.8*	5.0 ± 4.0	16.2 ± 8.6*	3.0 ± 3.3
Any [%]	31.0 ± 18.1*^,1^	4.7 ± 3.6	30.6 ± 16.0*	5.1 ± 4.2	17.8 ± 7.1*	2.2 ± 1.4
**FDS right**						
Tonic [%]	1.1 ± 1.2*	0.0 ± 0.0	1.1 ± 1.3	0.0 ± 0.0	1.3 ± 2.0	0.0 ± 0.0
Phasic [%]	20.6 ± 5.8*	3.0 ± 1.8	18.1 ± 7.0*	3.2 ± 1.7	16.0 ± 5.8*	2.0 ± 1.5
Any [%]	23.8 ± 6.4*^,1^	3.0 ± 1.8	20.4 ± 7.7*	3.2 ± 1.7	17.2 ± 6.0*	2.0 ± 1.5
**FDS left**						
Tonic [%]	2.7 ± 2.5*	0.0 ± 0.0	2.7 ± 3.3*	0.0 ± 0.0	1.9 ± 1.4*	0.0 ± 0.0
Phasic [%]	20.1 ± 7.4*	3.5 ± 1.9	20.2 ± 7.9*	3.5 ± 1.9	16.1 ± 6.1*	2.2 ± 1.4
Any [%]	24.1 ± 9.4*	3.5 ± 2.0	23.5 ± 10.0*	3.6 ± 1.9	17.8 ± 7.1*	2.2 ± 1.4
**TA right**						
Tonic [%]	0.3 ± 0.6	0.0 ± 0.0	0.4 ± 0.8	0.0 ± 0.0	0.5 ± 0.5	0.0 ± 0.0
Phasic [%]	13.5 ± 5.2^1^	10.2 ± 6.4	13.6 ± 10.1	6.7 ± 3.9	8.8 ± 4.6	6.7 ± 6.9
Any [%]	14.2 ± 5.4^1^	10.4 ± 6.3	14.4 ± 10.5	6.9 ± 3.7	9.2 ± 4.6	6.8 ± 6.9
**TA left**						
Tonic [%]	0.1 ± 0.3	0.0 ± 0.0	0.2 ± 0.6	0.0 ± 0.0	0.2 ± 0.3	0.0 ± 0.0
Phasic [%]	15.4 ± 6.3	11.4 ± 6.9	12.4 ± 5.2	7.6 ± 6.4	10.8 ± 5.7	5.9 ± 5.9
Any [%]	16.0 ± 6.5	11.8 ± 6.5^2^	12.9 ± 5.3	7.7 ± 6.4	11.1 ± 5.8	5.9 ± 5.9

*RBD* rapid eye movement sleep behaviour disorder, *FDS* flexor digitorum superficialis muscle, *TA* tibialis anterior muscle.

Statistics: *significant difference between RBD and controls, ^1^significant difference between RBDtector and novice scorer, no significant difference found between RBDtector and expert scorer and expert and novice scorer. Significance was accepted at p < 0.05 uncorrected, Student’s *t* tests and Mann–Whitney *U* tests were applied as appropriate.

**Figure 1:**
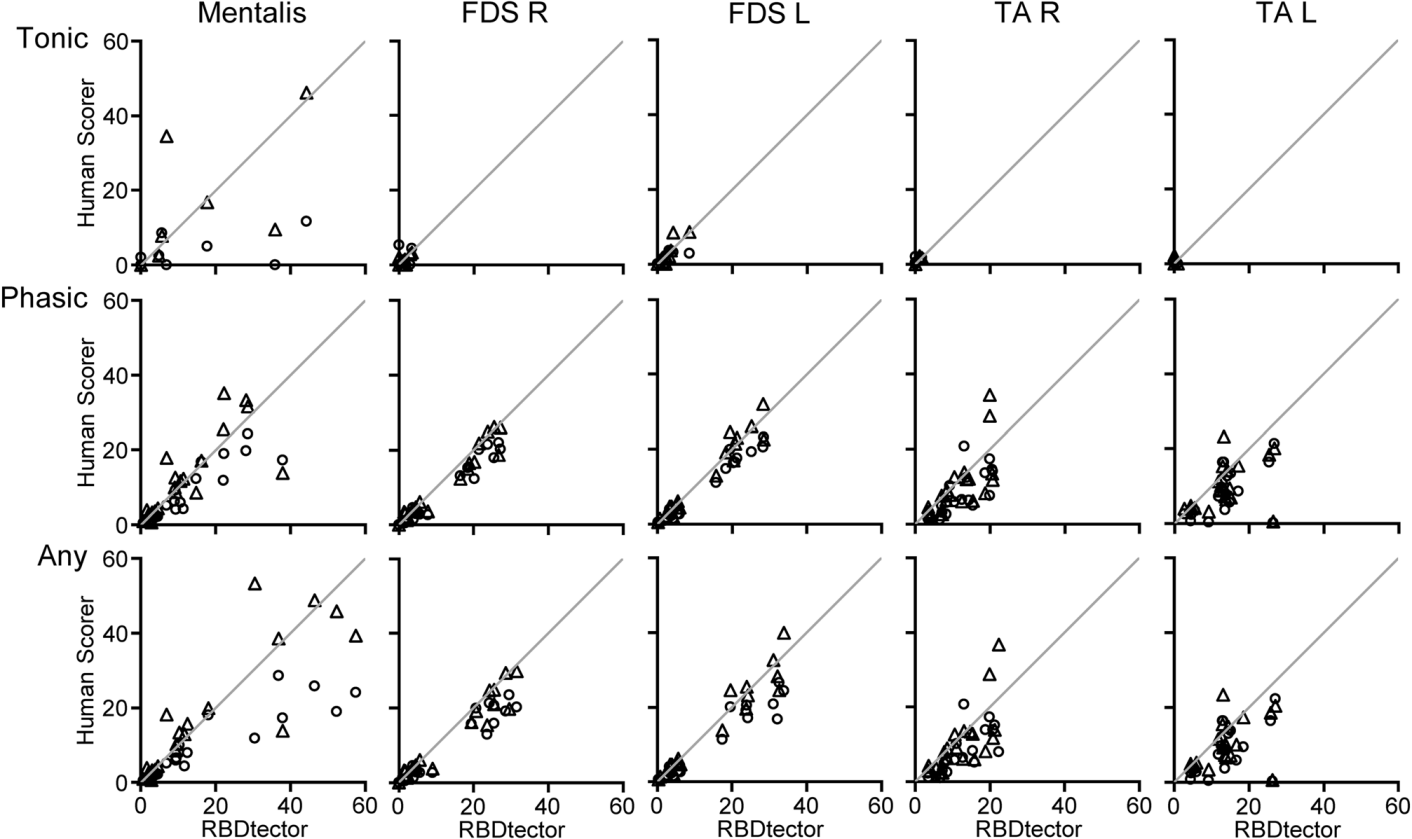
Correlation plots of the *RBDtector* scorings of the percentage of tonic, phasic, and any muscle activity in five different muscles during REM sleep against two human scorers (triangle, expert scorer and circles, novice scorer). Line of unity in light grey. *FDS* flexor digitorum superficialis muscle, *TA* tibialis anterior muscle, *R* right, *L* left.

At the subject level, we observed the highest agreement for phasic and any activity of the FDS between *RBDtector* and human scorings with R^2^ values above 0.9. Agreement of mentalis EMG scorings ranged from R^2^-values of 0.41/0.57 (tonic activity) to 0.82/0.75 (any activity) with a higher agreement between *RBDtector* and the expert scorer. Scorings of the TA EMG showed only minor agreement between *RBDtector* and human scorers (Fig. 1, Table 3).

**Table 3:** Metrics of agreement between *RBDtector* and human scorers.

	RBDtector versus	RBDtector versus	Expert versus
	Expert scorer	Novice scorer	Novice scorer
**Mentalis**			
Tonic	r = 0.76, R^2^ = 0.57, κ = 0.68	r = 0.64, R^2^ = 0.41, κ = 0.38	r = 0.67, R^2^ = 0.44, κ = 0.41
Phasic	r = 0.78, R^2^ = 0.61, κ = 0.65	r = 0.91, R^2^ = 0.83, κ = 0.59	r = 0.87, R^2^ = 0.76, κ = 0.55
Any	r = 0.87, R^2^ = 0.75, κ = 0.76	r = 0.90, R^2^ = 0.82, κ = 0.64	r = 0.82, R^2^ = 0.68, κ = 0.62
**FDS right**			
Tonic	r = 0.79, R^2^ = 0.62, κ = 0.63	r = 0.38, R^2^ = 0.15, κ = 0.58	r = 0.73, R^2^ = 0.53, κ = 0.61
Phasic	r = 0.97, R^2^ = 0.94, κ = 0.78	r = 0.98, R^2^ = 0.96, κ = 0.72	r = 0.96, R^2^ = 0.92, κ = 0.74
Any	r = 0.97, R^2^ = 0.93, κ = 0.83	r = 0.96, R^2^ = 0.93, κ = 0.78	r = 0.95, R^2^ = 0.91, κ = 0.78
**FDS left**			
Tonic	r = 0.90, R^2^ = 0.82, κ = 0.79	r = 0.83, R^2^ = 0.69, κ = 0.65	r = 0.78, R^2^ = 0.61, κ = 0.61
Phasic	r = 0.97, R^2^ = 0.94, κ = 0.80	r = 0.99, R^2^ = 0.97, κ = 0.72	r = 0.97, R^2^ = 0.94, κ = 0.75
Any	r = 0.97, R^2^ = 0.94, κ = 0.84	r = 0.97, R^2^ = 0.93, κ = 0.78	r = 0.95, R^2^ = 0.90, κ = 0.80
**TA right**			
Tonic	r = 0.94, R^2^ = 0.88, κ = 0.80	r = 0.67, R^2^ = 0.44, κ = 0.57	r = 0.62, R^2^ = 0.38, κ = 0.50
Phasic	r = 0.48, R^2^ = 0.70, κ = 0.65	r = 0.74, R^2^ = 0.55, κ = 0.61	r = 0.54, R^2^ = 0.30, κ = 0.65
Any	r = 0.72, R^2^ = 0.52, κ = 0.67	r = 0.74, R^2^ = 0.55, κ = 0.63	r = 0.54, R^2^ = 0.29, κ = 0.67
**TA left**			
Tonic	r = n/a, R^2^ = n/a, κ = n/a	r = n/a, R^2^ = n/a, κ = n/a	r = n/a, R^2^ = n/a, κ = n/a
Phasic	r = 0.45, R^2^ = 0.20, κ = 0.67	r = 0.54, R^2^ = 0.29, κ = 0.61	r = 0.88, R^2^ = 0.78, κ = 0.70
Any	r = 0.45, R^2^ = 0.20, κ = 0.68	r = 0.53, R^2^ = 0.29, κ = 0.62	r = 0.87, R^2^ = 0.75, κ = 0.71

*iRBD* idiopathic rapid eye movement sleep behaviour disorder, *FDS* flexor digitorum superficialis muscle, *TA* tibialis anterior muscle, *n/a* not applicable (not enough events).

At the single epoch level, concordance between human scorers and *RBDtector* was substantial for most EMG channels as indicated by Cohen’s κ values above 0.6. Again, the highest agreement was obtained for phasic and any activity of the FDS (Table 3), and agreement of *RBDtector* results was higher with the human expert (all κ values > 0.63) than with the novice (lowest agreement for tonic activity at mentalis EMG, κ = 0.38). The concordance of *RBDtector* results with the human expert was higher than the concordance between human scorers.

### Performance of RBDtector

In a second step, we evaluated 174 PSGs including 72 PSGs of subjects without RBD and 102 with RBD by *RBDtector*. Demographics, PSG characteristics, and RSWA indices are summarized in Table 4. For better readability, we only present merged left and right RSWA indices of FDS and TA EMG and reported only the combination of mentalis and FDS EMG with the highest discriminatory performance (= mentalis any + FDS bilateral any) as well as the combination originally proposed by the SINBAR group (= mentalis any + FDS bilateral phasic).

**Table 4:** Demographics, polysomnography data, and RSWA metrics of subjects evaluated by *RBDtector*.

Total n = 174	Controls n = 56	PD noRBD n = 16	iRBD n = 81	PD + RBD n = 21	p-value
**Demographics**					
Age [y]	62.1 ± 11.2^2,3^	64.4 ± 8.3	66.9 ± 6.4	68.4 ± 6.7	**0.031**^**§**^
Sex [m/f]	42/14	13/3	69/12	14/7	0.220^#^
BMI [kg/m^2^]	25.8 ± 0.0	28.1 ± 4.6	25.3 ± 3.3	25.1 ± 4.3	0.068^§^
**Polysomnography**					
Total bedtime [min]	457.6 ± 66.0	440.6 ± 71.8	465.3 ± 66.7	472.2 ± 63.5	0.273^§^
Sleep efficiency [%]	82.3 ± 11.7	77.1 ± 22.2	82.3 ± 9.5	81.3 ± 12.9	0.965^§^
Sleep latency N2 [min]	15.2 ± 15.5^3^	28.5 ± 70.7^2^	18.4 ± 15.6^3^	8.7 ± 7.4	** < 0.001**^**§**^
N1 [min]	76.2 ± 46.6	63.1 ± 31.2	78.7 ± 41.7	62.7 ± 24.1	0.385^§^
N2 [min]	180.0 ± 50.7	171.9 ± 84.7	173.8 ± 43.4	189.9 ± 61.3	0.604*
SWS [min]	56.2 ± 26.1	57.1 ± 40.9	58.7 ± 26.6	52.8 ± 36.1	0.865*
REM [min]	59.3 ± 24.9	47.8 ± 30.7	59.3 ± 23.2	69.1 ± 40.0	0.132*
AHI [/h]	13.3 ± 13.7^2^	16.1 ± 14.7^2^	7.1 ± 9.5	11.4 ± 11.9	**0.008**^**§**^
PLMSI [/h]	35.4 ± 39.2	36.9 ± 49.0	45.4 ± 41.4	28.6 ± 29.5	0.051^§^
**RSWA metrics (removal of arousals, snoring and technical artefacts)**					
Mentalis, tonic	0.3 ± 1.1^2,3^	0.0 ± 0.0^2,3^	10.4 ± 12.7	14.9 ± 12.8	** < 0.001**^**§**^
Mentalis, phasic	5.7 ± 3.7^2,3^	4.6 ± 2.7^2,3^	23.6 ± 11.3	25.8 ± 7.8	** < 0.001**^**§**^
Mentalis, any	6.0 ± 4.3^2,3^	4.7 ± 2.7^2,3^	33.9 ± 18.7	40.8 ± 17.0	** < 0.001**^**§**^
FDS bilat, tonic	0.1 ± 0.3^2,3^	0.1 ± 0.3^2^	7.0 ± 8.4	4.9 ± 7.3	** < 0.001**^**§**^
FDS bilat, phasic	7.4 ± 4.7^2,3^	4.9 ± 4.1^2,3^	33.5 ± 11.3^3^	25.6 ± 8.8	** < 0.001**^**§**^
FDS bilat, any	7.5 ± 4.6^2,3^	5.1 ± 4.2^2,3^	39.1 ± 15.0	31.4 ± 13.6	** < 0.001**^**§**^
TA bilat, tonic	0.3 ± 0.8^1,2,3^	2.6 ± 4.1	1.7 ± 3.0	2.4 ± 5.6	** < 0.001**^**§**^
TA bilat, phasic	20.1 ± 12.5^2,3^	18.3 ± 9.7^2,3^	29.9 ± 12.3	25.9 ± 9.9	** < 0.001**^**§**^
TA bilat, any	20.5 ± 12.5^2,3^	21.7 ± 13.4^2^	31.3 ± 12.9	28.9 ± 14.1	** < 0.001**^**§**^
Mentalis, any + FDS, any	11.2 ± 4.1^2,3^	10.1 ± 5.9^2,3^	54.2 ± 18.5	51.5 ± 15.3	** < 0.001**^**§**^
Mentalis, any + FDS, phasic	11.1 ± 4.0^2,3^	9.9 ± 5.7^2,3^	51.4 ± 17.2	49.0 ± 14.8	** < 0.001**^**§**^
**RSWA metrics (removal of arousals events only)**					
Mentalis, tonic	0.7 ± 1.9^2,3^	0.4 ± 1.5^2,3^	10.8 ± 13.2	13.7 ± 12.5	** < 0.001**^**§**^
Mentalis, phasic	8.9 ± 7.5^2,3^	6.7 ± 5.5^2,3^	24.8 ± 10.6	25.7 ± 7.5	** < 0.001**^**§**^
Mentalis, any	9.7 ± 8.3^2,3^	7.2 ± 6.3^2,3^	35.4 ± 17.9	39.4 ± 16.1	** < 0.001**^**§**^
FDS bilat, tonic	0.3 ± 1.5^2,3^	0.2 ± 0.3^2,3^	7.1 ± 8.4	11.4 ± 13.7	** < 0.001**^**§**^
FDS bilat, phasic	9.0 ± 8.4^2,3^	8.0 ± 8.0^2,3^	33.2 ± 11.3^3^	25.2 ± 8.7	** < 0.001**^**§**^
FDS bilat, any	9.3 ± 8.6^2,3^	8.3 ± 8.3^2,3^	38.8 ± 15.0	36.8 ± 14.8	** < 0.001**^**§**^
TA bilat, tonic	0.7 ± 3.2^1,2,3^	2.4 ± 4.0	2.1 ± 5.3	2.4 ± 5.5	** < 0.001**^**§**^
TA bilat, phasic	21.1 ± 13.8^2^	18.0 ± 9.5^2^	31.3 ± 14.0	24.7 ± 10.3	** < 0.001**^**§**^
TA bilat, any	22.1 ± 14.8^2,3^	21.2 ± 13.1^2^	32.9 ± 15.0	27.7 ± 14.2	** < 0.001**^**§**^
Mentalis, any + FDS, any	18.1 ± 11.8^2,3^	15.9 ± 10.9^2,3^	56.6 ± 17.7	57.6 ± 16.1	** < 0.001**^**§**^
Mentalis, any + FDS, phasic	17.9 ± 11.7^2,3^	15.7 ± 10.8^2,3^	53.8 ± 16.4	50.1 ± 14.3	** < 0.001**^**§**^

*BMI* body mass index, *N1/2* non-rapid eye movement sleep stage 1/2, *SWS* slow wave sleep, *REM* rapid eye movement sleep, *AHI* apnoea hypopnea index, *PLMSI* periodic limb movements in sleep index.

Statistics: *ANOVA, ^§^Kruskal Wallis test (post-hoc testing with Mann Whitney *U* test: ^1^different to PD noRBD, ^2^different to iRBD, ^3^different to PD + RBD at p < 0.05 uncorrected), ^#^Chi square test.

Significant values are in bold.

We analysed RSWA indices after removing arousal events, mentalis EMG channels affected by snoring artefacts, and EMG channels corrupted by technical artefacts. That reassessment led to the exclusion of mentalis EMG channels in 29 PSGs (= 16.7% of all recordings), FDS EMG channels in 8 PSGs (= 6.3% of recordings—only 143 PSGs included FDS recordings), and TA EMG channels in 11 PSGs (= 6.3% of all recordings).

#### RBDtector results with the removal of REM (mini) epochs containing arousals, snoring, and technical artefacts

**Figure 2:**
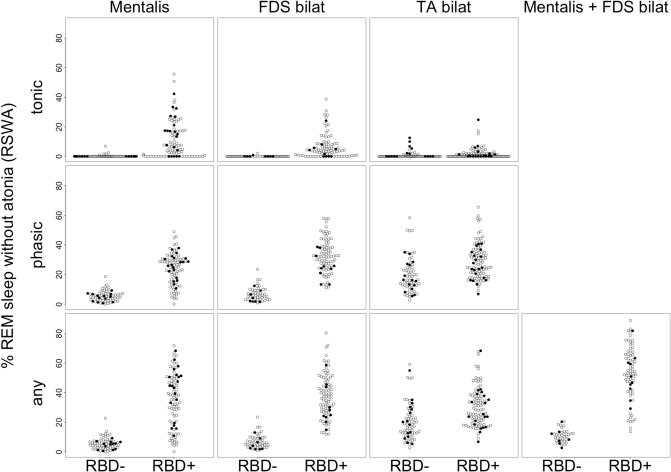
Dot plots of individual tonic (upper row), phasic (middle row), and (any) percentage of rapid eye movement (REM) sleep without atonia after removal of arousals, snoring and technical artefacts in mentalis EMG and combination of FDS and TA EMG channels of both sides as well as the combination of mentalis EMG and combined FDS EMG channels. Subjects without RBD (RBD-, patients with Parkinson’s disease marked as black dots) are presented on the left, and subjects with RBD on the right (RBD +). *FDS* flexor digitorum superficialis muscle, *TA* tibialis anterior muscle, *R* right, *L* left.

**Figure 3:**
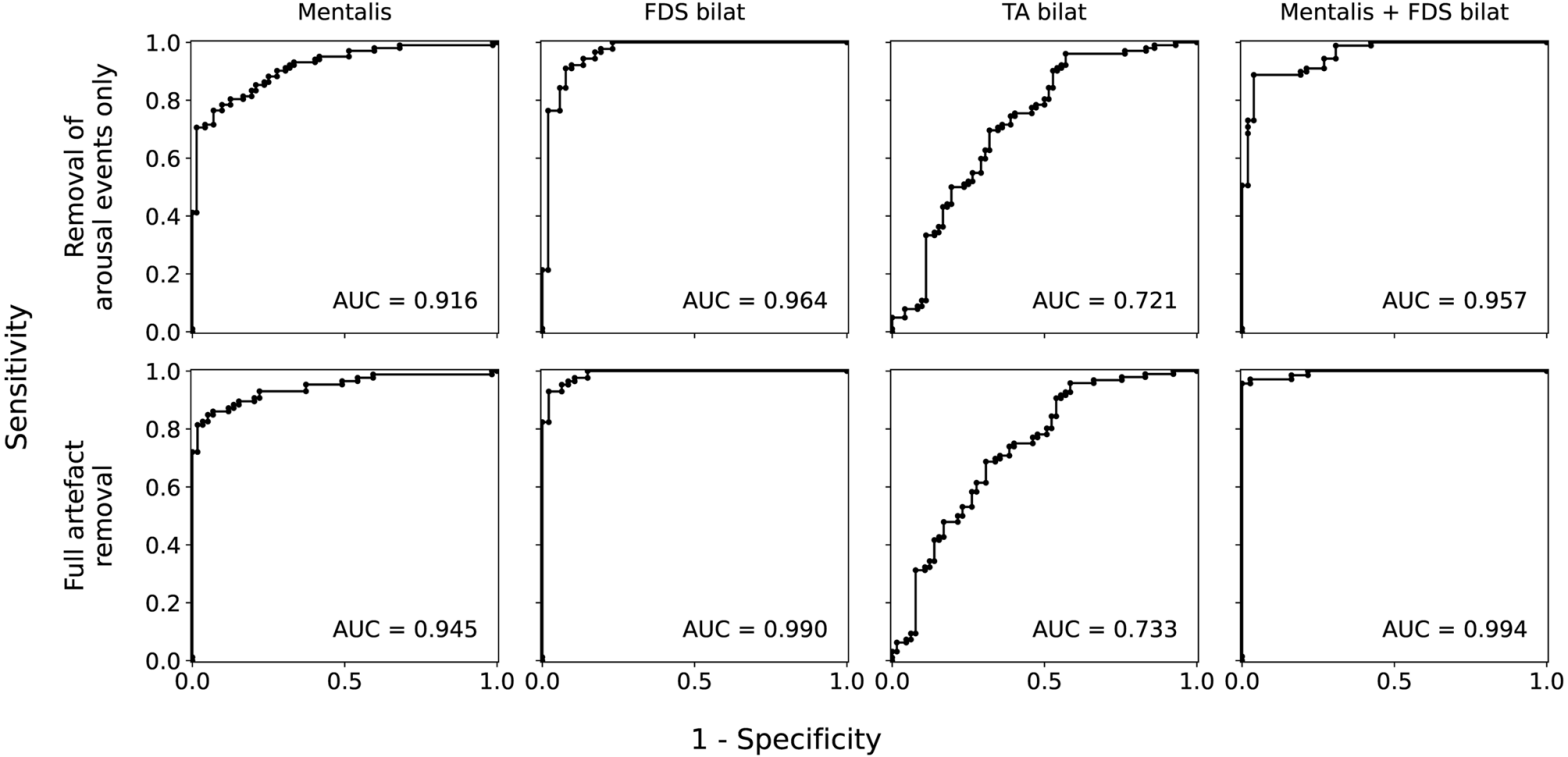
Receiver operating characteristic (ROC) curves of selected electromyography channels: from left to right: mentalis, any; flexor digitorum superficialis (FDS) bilateral (bilat), any; tibialis anterior (TA) bilateral, any; combination of mentalis, any + FDS, any. Upper row provides ROC curves of *RBDtector* performance after removal of arousals only and lower row provides ROC curves after removal of arousals, snoring and technical artefacts (“full artefact removal”).

In addition to Table 4, Fig. 2 illustrates the distribution of the amount of RSWA within the groups. After merging the subjects without RBD and subjects with RBD, RBD was associated with significantly elevated muscle activity in all EMG channels examined (all p-values < 0.003, Mann–Whitney *U* tests). Table 5 summarizes the accuracy metrics of *RBDtector* at 100% specificity and selected ROC curves are provided in Fig. 3. Estimations of RSWA with FDS EMG were superior in AUC, sensitivity, and accuracy to mentalis EMG (Table 5). A combination of both mentalis and FDS EMG channels provided the highest performance, specifically, the combination of any activity of the mentalis plus any activity of the FDS with 96% sensitivity (at 100% specificity) and 97% accuracy at a cut-off of 20.6% RSWA (Table 5). Applying the previously published cut-off value of 32% from the SINBAR group of any activity of the mentalis phasic plus phasic activity of the FDS^18^, *RBDtector* had a sensitivity of 86% (at 100% specificity). Additional metrics at the original SINBAR cut-offs are given in Supplementary Table 1.

**Table 5:** Metrics of the accuracy of *RBDtector*.

	AUC	Cut-off value with 100% specificity	Sensitivity at 100% specificity	Accuracy at 100% specificity
**Removal of arousals, snoring and technical artefacts**				
Individual muscles				
Mentalis, tonic	0.848	7.1	50%	70%
Mentalis, phasic	0.942	19.0	71%	83%
Mentalis, any	0.945	23.5	72%	83%
FDS bilat, tonic	0.885	2.2	66%	78%
FDS bilat, phasic	0.989	23.7	79%	86%
FDS bilat, any	0.990	23.8	82%	89%
TA bilat, tonic	0.673	13.9	3%	42%
TA bilat, phasic	0.739	59.0	2%	42%
TA bilat, any	0.733	62.5	3%	42%
Muscle combinations				
Mentalis, any + FDS, any	0.994	20.6	96%	97%
Mentalis, any + FDS, phasic	0.993	20.6	96%	97%
**Removal of arousal events only**				
Individual muscles				
Mentalis, tonic	0.833	10.4	41%	66%
Mentalis, phasic	0.902	36.8	13%	48%
Mentalis, any	0.916	41.5	41%	66%
FDS bilat, tonic	0.876	9.8	25%	52%
FDS bilat, phasic	0.959	51.8 (27.1)	6% (69%)	40% (80%)
FDS bilat, any	0.964	51.3 (28)	21% (76%)	50% (85%)
TA bilat, tonic	0.688	24.3	2%	43%
TA bilat, phasic	0.732	59.3	3%	43%
TA bilat, any	0.721	63.9	5%	44%
Muscle combinations				
Mentalis, any + FDS, any	0.957	59.2 (48.8)	51% (73%)	69% (83%)
Mentalis, any + FDS, phasic	0.952	59.2 (48.7)	37% (67%)	60% (79%)

*AUC* area under the curve, *bilat* bilateral, *FDS* flexor digitorum superficialis muscle, *TA* tibialis anterior muscle.

Values in parentheses represent cut-off values after the exclusion of a single RBD-negative subject, which exhibited technical artefacts on both FDS channels (example given in Figure s1).

Poorest performance was observed with TA EMG estimations of RSWA, and PLMI correlated significantly with phasic and any activity of the TA (rho > 0.4, p < 0.001).

#### RBDtector results with removal of arousals only

**Figure 4:**
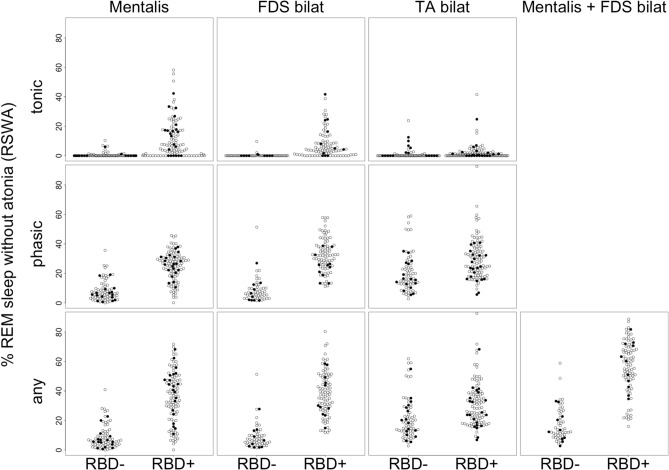
Dot plots of individual tonic (upper row), phasic (middle row), and (any) percentage of rapid eye movement (REM) sleep without atonia after removal of arousals only in mentalis EMG and combination of FDS and TA EMG channels of both sides as well as the combination of mentalis EMG and combined FDS EMG channels. Subjects without RBD (RBD-, patients with Parkinson’s disease marked as black dots) are presented on the left, and subjects with RBD on the right (RBD+). *FDS* flexor digitorum superficialis muscle, *TA* tibialis anterior muscle, *R* right, *L* left.

In addition to Table 4, Fig. 4 illustrates the distribution of the amount of RSWA within the groups, when only REM (mini-) epochs containing arousals were discarded. After merging the subjects without RBD and subjects with RBD, RBD was associated with significantly elevated muscle activity in all studied EMG channels (all p-values < 0.001, Mann–Whitney *U* tests). Table 5 summarizes the accuracy metrics of *RBDtector* at 100% specificity. Apart from one outlier with overt technical artefacts (example given in Fig. s1), estimations of RSWA with FDS EMG were again superior in AUC, sensitivity, and accuracy compared to mentalis EMG (Table 5). A combination of both mentalis and FDS EMG channels provided comparable performance to FDS EMG alone (Table 5). ROC curves are provided in Fig. 3.

### Comparison of amplitude and duration of muscle activity

Subjects with RBD showed not only an increased amount of RSWA but bouts of non-tonic activity (= phasic and any activity) had higher amplitudes in all EMG channels (subjects without RBD vs. with RBD: mentalis, 11.8 ± 4.7 mV vs. 15.5 ± 4.5 mV; FDS right, 21.0 ± 10.6 mV vs. 31.3 ± 12.2 mV; FDS left, 19.4 ± 8.5 mV vs. 29.8 ± 11.0 mV, TA right, 30.0 ± 15.5 mV vs. 43.6 ± 18.6 mV; TA left, 30.5 ± 14.7 mV vs. 43.5 ± 18.6 mV; all p < 0.001, Mann–Whitney *U* tests). AUC to discriminate between RBD-positive and -negative subjects was below 0.8 for all EMG channels.

Similarly, phasic muscle bouts showed longer duration in the context of RBD (subjects without RBD vs. subjects with RBD: mentalis, 614.5 ± 218.0 ms vs. 941.5 ± 227.5 ms; FDS right, 514.2 ± 221.3 ms vs. 954.4 ± 199.7 ms; FDS left, 512.0 ± 206.0 ms vs. 914.3 ± 176.1 ms, TA right, 501.8 ± 220.1 ms vs. 667.3 ± 208.8 ms; TA left, 578.9 ± 291.6 ms vs. 646.6 ± 187.8 ms; all p < 0.003, Mann–Whitney *U* tests). AUC to discriminate between RBD-positive and -negative subjects was below 0.8 for both TA channels, 0.87 for mentalis, and 0.943 for FDS right as well as 0.935 for FDS left.

### RSWA estimations of RBDtector on a previously published dataset

We also evaluated the performance of *RBDtector* on the same dataset where a commercially available integrated software, which scores according to the SINBAR criteria, has been previously validated^25^. This dataset included 20 patients with RBD and 60 controls. *RBDtector* was not applied to 3 recordings from controls due to technical issues (one had fragmented REM sleep, and two EDF files were discontinuous). In all recordings, muscular activity related to manually scored arousals was removed. To allow a fair comparison with the previous manual and automatic scorings performed in this dataset, *RBDtector* processed the data without any further artefact management at a sampling frequency of 1000 Hz with a bandpass filter between 50 and 300 Hz and a 48 to 52 Hz notch filter, which were applied in the previous study. *RBDtector* showed comparable quantifications of RSWA with the SINBAR sleep expert (Spearman rho values ranging from 0.62 to 0.96, best comparability with scorings of the FDS EMG, see Fig. s2). These values are similar to the correlation achieved by the integrated software used by SINBAR^25^. When using the thresholds proposed by the SINBAR group^18^, *RBDtector* achieved RBD classification sensitivity and specificity in the 95% confidence intervals of the ones achieved by the commercially available validated software (Table 6).

**Table 6:** Comparison of performance detection for RBD achieved in a previously published dataset^25^.

	Performance	Manual^25^	Automatic without artefact correction^25^	RBDtector without artefact removal
Mentalis, phasic	Sensitivity	0.90 (0.68–0.99)	0.90 (0.68–0.99)	0.80
	Specificity	0.68 (0.55–0.79)	0.47 (0.33–0.60)	0.40
Mentalis, tonic	Sensitivity	0.64 (0.38–0.82)	0.75 (0.51–0.91)	0.80
	Specificity	1.00 (0.92–1.00)	0.98 (0.91–1.00)	0.93
Mentalis, any	Sensitivity	0.85 (0.62–0.97)	0.90 (0.68–0.99)	0.85
	Specificity	0.73 (0.60–0.84)	0.45 (0.32–0.58)	0.39
FDS, phasic	Sensitivity	0.94 (0.73–1.00)	0.95 (0.72–1.00)	0.94
	Specificity	0.87 (0.75–0.94)	0.81 (0.69–0.90)	0.82
Mentalis, any + FDS, phasic	Sensitivity	0.83 (0.59–0.96)	0.94 (0.73–1.00)	0.83
	Specificity	0.87 (0.75–0.94)	0.72 (0.59–0.83)	0.60

The data obtained from the publication of Frauscher et al.^25^ are shown as mean and 95% confidence interval.

## Discussion

We introduce *RBDtector*, an open-source software algorithm to quantify RSWA following the established SINBAR scoring scheme. We compared the *RBDtector* scorings to human evaluations and tested its performance on a large dataset. *RBDtector* showed robust comparability to human scorings with the best performance for EMG analysis of the flexor digitorum superficialis. Furthermore, the concordance of *RBDtector* results with the human expert was higher than the agreement of the less trained human scorer with the sleep expert. Even in the absence of artefact removal, *RBDtector* could detect RBD-positive subjects with high accuracy by combining RWSA indices of the mentalis and flexor digitorum superficialis in our dataset. RBD-positive subjects not only showed a higher quantity of muscle activity, but activity bouts also displayed higher amplitude and duration on average. Eventually, *RBDtector* showed high comparability with sleep expert scorings in an independent dataset and could identify RBD patients with similar sensitivity and specificity to a comparable commercial integrated software^25^.

### Relevance of selected muscles to detect RSWA

The SINBAR criteria for RSWA is the only scoring scheme that considers muscles of the face, arms, and legs^18,28,29^. We selected this scheme to be implemented in *RBDtector* to allow for high flexibility in the analysis of EMG channels. Additionally, as already reported in the initial SINBAR publication^18,20^ and recently supported by the new guidelines from the International RBD Study Group^17^, we confirmed the usefulness of adding arm EMG to the standard PSG montage for detection of RBD in our large sample. FDS EMG was beneficial in multiple ways: (i) these channels showed the lowest frequency of artificial signals compared to mentalis and TA EMG, (ii) the congruency of human scorings among each other and with *RBDtector* was the highest, and (iii) the discriminatory value to detect RBD was superior to all other individual channels for the quantity of RSWA and for metrics of single activity bouts (i.e. amplitude and duration). Accordingly, we strongly concur with the recommendations to use arm EMG when screening for or evaluating RBD in subjects^21^. This recommendation is further supported by the high specificity, sensitivity and accuracy provided by the combination of RSWA estimations from mentalis and FDS EMG (i.e. mentalis any + FDS any), which was superior to classification performance when only EMG channels from a standard PSG montage were used.

Oppositely, the assessment of leg EMG, i.e. TA EMG, as part of the standard PSG montage, did not add relevant information to discriminate RBD-positive subjects on an individual level, even though TA activity was higher in RBD-positive subjects on a group level. TA EMG might especially be confounded by (periodic) limb movements unrelated to RSWA in RBD^30^. Correspondingly, we found a positive correlation between PLMI and phasic as well as any activity of TA.

Hitherto, studies on RSWA primarily focused on the evaluation of increased muscle activity on the chin^17,23,24,28,29,31,32^. The high relevance of chin EMG is also displayed by the fact that the highest accuracy to detect RBD-positive subjects was achieved when combining RSWA metrics of the chin and arms consistent with the recommendations in the initial SINBAR publication^18^. However, the quantification of chin EMG might potentially be affected by artefacts, which could lead to biased estimates of RSWA.

### Relevance of artefact management

A thorough artefact removal did increase the accuracy of *RBDtector* results in our analysis. However, a current consensus on optimal artefact management is lacking^17^. Chin EMG is highly prone to a wide variety of different artefacts: snoring, air flow-mediated movements of the lips/chin, ECG artefacts, and electrode dysfunction in bearded subjects. Furthermore, in our comparison of two human scorers, congruency was poor on an individual epoch level, which is in line with a previous study^20^. We, therefore, decided to discard the complete EMG channel instead of selected epochs from the analysis if an extensive amount of potential artefacts were present. This conveniently shortens the manual labour for artefact elimination, as the decision for channel exclusion can be made during routine PSG evaluation. However, this rigorous step leads to the exclusion of the chin EMG in many subjects.

Particularly, when evaluating RSWA to detect iRBD patients, who are mostly male and older, the likelihood of sleep-related breathing disorders is increased^33^, which impacts the occurrence of chin EMG artefacts^20^. This might implicate false RSWA estimations and subsequently potential misclassification. Surprisingly, normative data for RSWA in subjects with AHI ≥ 15/hour are sparse^18,28,29,31,34^, even though an AHI of 15.5/hour is considered normal in healthy adults at the age of 65–79 years according to a recent meta-analysis^33^. This is, however, precisely the age span to expect, when screening for RBD in the context of α-synucleinopathies as neurodegenerative disorders are classical age-related diseases showing increasing prevalence and incidence in the ageing population. Interestingly, we also found a correlation of age with RSWA in the FDS in RBD-positive and RBD-negative subjects. This finding needs further validation in future studies.

We did not exclude subjects by a given AHI cut-off. Hence, our data likely depict clinical reality. At best, an automated artefact detection system covering multiple potential artefacts at different EMG channels (e.g. snoring, respiration, and technical malfunction) should be implemented on a mini-epoch level. However, such a system was out of the scope of our current study. Including machine-learning approaches or adding information from acoustic recordings might help to specifically reduce the influence of snoring artefacts on RSWA quantification^35,36^. As for the modular structure of *RBDtector*, the future addition of such an artefact-detection module to improve the interpretability of mentalis EMG is feasible and might be desirable for sleep laboratories without access to arm muscle EMG.

The exclusion of artefacts on the other EMG channels, i.e. FDS, was less relevant as their frequency was considerably lower and artefacts mostly stemmed from obvious technical dysfunction of the EMG channel with a complete lack of interpretability for the channel.

### Comparison to other computerized RSWA detection algorithms

Visual scoring of RSWA is still the gold standard of RSWA quantification^17,19^. Accordingly, we aimed to implement an established visual scoring scheme including various EMG channels in *RBDtector*. Multiple visual scorings schemes have been introduced by several groups with high expertise in RSWA and RBD assessments^18,28,29^, and the SINBAR system as well as the criteria introduced by McCarter and colleagues were implemented in commercial software solutions^23,25^. However, SINBAR criteria exclusively are included in the AASM scoring manual, thus likely constituting the most widely used in clinical practice. Additionally, SINBAR is the only visual scoring scheme validated on arm EMG channels.

In contrast, multiple computerized RSWA quantifications including fully-automated and semi-automated algorithms exist, which show moderate comparability between each other^24,32,34,37^. These algorithms, however, mostly lack the validation on arm EMGs, which are less prone to artefact contamination than the mentalis EMG^32,38^, especially in patients with airway-related sleep disorders. As artefact management is a big distinguishing factor in such automated quantifications^20,31^, direct comparison without arm EMG is often inconclusive. In a recent comparative study on six different algorithms^24^, sensitivity, specificity, and accuracy were in the range of 60–70%, which is lower than the metrics we could obtain with *RBDtector* when applying a brief elimination of severely artefact-affected EMG channels. The detection of RSWA events of all SINBAR categories, as opposed to approaches that only classify whether a subject has RSWA or not, facilitates further research on differences in disease characteristics depending on specific EMG presentations. More direct comparative research on the different quantitative RSWA detection algorithms is necessary to achieve a fully conclusive view. Meanwhile, modern machine-learning approaches and the inclusion of arm EMG might overcome existing caveats of previous automated RSWA algorithms in the future.

The second goal of *RBDtector* was to provide a software tool free of charge with open-source code and the possibility to modify the tool to centre-specific conditions. This significantly distinguishes *RBDtector* from previously published computerized solutions for RSWA estimation. We aimed for a computer algorithm with a modular architecture with separated import and analyses modules to be readily customized to import data of varying systems and hope that this unique combination will facilitate the use and future adaptation of *RBDtector.*

### Limitations

Our sample for initial validation of human and *RBDtector* scorings comprised only 10 PSGs of subjects with and 10 PSGs without RBD. Still, more than 20,000 mini epochs were analysed by two independent human scorers, which should provide sufficient data to estimate the parameters of *RBDtector’s* accuracy. Additionally, we could compare *RBDtector* scorings to an independent, previously published dataset, and could achieve high comparability to human scorings comparable to a commercial software solution^25^. However, the congruency among human scorers and compared to *RBDtector* was only modest for tonic activity of the chin. This finding is in line with a recent report evaluating the inter-scorer agreement of tonic and phasic activity assessment, showing considerable differences among scorers, especially for tonic activity^22^. Hence, all estimations of tonic activity on the chin have a high intrinsic variability even when artefacts are handled carefully.

Our RBD-positive subjects were all most likely affected by an α-synucleinopathy, and we did not test *RBDtector* performance in other disorders related to RBD and RSWA. Future studies are warranted to evaluate *RBDtector* for these entities. Finally, *RBDtector* is currently optimized to one commercial PSG system and using it with another system might imply minor changes in the code to import sleep stages and EMG data. However, due to its modular architecture, the RSWA-scoring part of *RBDtector* does not need to be changed. We provide thorough annotations in the supplementary material to facilitate importing other formats.

### Conclusion and outlook

*RBDtector* is an open-source tool to quantify RSWA according to an established visual scoring scheme and can separate RBD-positive from RBD-negative subjects with high accuracy—even in the presence of potential artefacts. We hope that this tool will facilitate RSWA quantification for researchers and might allow for higher comparability of RSWA quantifications between different centres as potential human bias can be minimized. As *RBDtector* is open-source and has a modular architecture, researchers may further customize it, e.g. including automated artefact detection, which is still an unsolved issue hampering the reliability and comparability of RSWA estimates.

## Supplementary Information

Supplementary Information.

## Data Availability

Data are available from the corresponding author upon reasonable request. Because the subject’s consent is required for data disclosure, we may disclose data conditionally through internal discussions, contact with the subjects, and the local ethical committee.

## References

[1] Haba-Rubio J (2018). Prevalence and determinants of rapid eye movement sleep behavior disorder in the general population. Sleep.

[2] Högl B (2022). Rapid eye movement sleep behaviour disorder: Past, present and future. J. Sleep Res..

[3] Dauvilliers Y (2018). REM sleep behaviour disorder. Nat. Rev. Dis. Primers.

[4] Miglis MG (2021). Biomarkers of conversion to α-synucleinopathy in isolated rapid-eye-movement sleep behaviour disorder. Lancet Neurol..

[5] Postuma RB (2019). Risk and predictors of dementia and parkinsonism in idiopathic REM sleep behaviour disorder: A multicentre study. Brain.

[6] Knudsen K (2018). In-vivo staging of pathology in REM sleep behaviour disorder: A multimodality imaging case-control study. Lancet Neurol..

[7] Heinzel S (2019). Update of the MDS research criteria for prodromal Parkinson’s disease. Mov. Disord..

[8] Sommerauer M (2014). Revisiting the impact of REM sleep behavior disorder on motor progression in Parkinson’s disease. Parkinsonism Relat. Disord..

[9] Fereshtehnejad SM (2015). New clinical subtypes of Parkinson disease and their longitudinal progression a prospective cohort comparison with other phenotypes. JAMA Neurol..

[10] Sommerauer M (2018). Evaluation of the noradrenergic system in Parkinson’s disease: An 11 C-MeNER PET and neuromelanin MRI study. Brain.

[11] Horsager J (2020). Brain-first versus body-first Parkinson’s disease: A multimodal imaging case-control study. Brain.

[12] Doppler CEJ (2021). Microsleep disturbances are associated with noradrenergic dysfunction in Parkinson’s disease. Sleep.

[13] Horsager J, Knudsen K, Sommerauer M (2022). Clinical and imaging evidence of brain-first and body-first Parkinson’s disease. Neurobiol. Dis..

[14] Borghammer P (2021). Neuropathological evidence of body-first vs. brain-first Lewy body disease. Neurobiol. Dis..

[15] Bedard MA (2019). Brain cholinergic alterations in idiopathic REM sleep behaviour disorder: A PET imaging study with 18 F-FEOBV. Sleep Med..

[16] Nepozitek J (2019). Simultaneous tonic and phasic REM sleep without atonia best predicts early phenoconversion to neurodegenerative disease in idiopathic REM sleep behavior disorder. Sleep.

[17] Cesari M (2021). Video-polysomnography procedures for diagnosis of rapid eye movement sleep behavior disorder (RBD) and the identification of its prodromal stages: Guidelines from the International RBD Study Group. Sleep.

[18] Frauscher B (2012). Normative EMG Values during REM Sleep for the diagnosis of REM sleep behavior disorder. Sleep..

[19] Berry, R. et al. The AASM manual for the scoring of sleep and associated events: rules, terminology and technical specifications: Version 2.6. Darien, IL: American Academy of Sleep Medicine Title. (2020).

[20] Cesari M (2021). Flexor digitorum superficialis muscular activity is more reliable than mentalis muscular activity for rapid eye movement sleep without atonia quantification: A study of interrater reliability for artifact correction in the context of semiautomated scoring of rapid eye movement sleep without atonia. Sleep.

[21] Cesari M (2022). Automatic analysis of muscular activity in the flexor digitorum superficialis muscles: A fast screening method for rapid eye movement sleep without atonia. Sleep.

[22] Bliwise DL (2018). Inter-rater agreement for visual discrimination of phasic and tonic electromyographic activity in sleep. Sleep.

[23] Jeppesen J (2018). Observations on muscle activity in REM sleep behavior disorder assessed with a semi-automated scoring algorithm. Clin. Neurophysiol..

[24] Cesari M (2018). Comparison of computerized methods for rapid eye movement sleep without atonia detection. Sleep.

[25] Frauscher B (2014). Validation of an integrated software for the detection of rapid eye movement sleep behavior disorder. Sleep.

[26] Postuma RB (2015). MDS clinical diagnostic criteria for Parkinson’s disease. Mov. Disord..

[27] Darien, I. The international classification of sleep disorders (ICSD-3). Am. Acad. Sleep Med. (2014).

[28] McCarter SJ (2014). Diagnostic thresholds for quantitative REM sleep phasic burst duration, phasic and tonic muscle activity, and REM atonia index in REM sleep behavior disorder with and without comorbid obstructive sleep apnea. Sleep..

[29] Lapierre O, Montplaisir J (1992). Polysomnographic features of REM sleep behavior disorder: Development of a scoring method. Neurology.

[30] Fantini ML, Michaud M, Gosselin N, Lavigne G, Montplaisir J (2002). Periodic leg movements in REM sleep behavior disorder and related autonomic and EEG activation. Neurology.

[31] Ferri R (2010). Improved computation of the atonia index in normal controls and patients with REM sleep behavior disorder. Sleep Med..

[32] Cesari M (2019). Validation of a new data-driven automated algorithm for muscular activity detection in REM sleep behavior disorder. J. Neurosci. Methods.

[33] Boulos MI (2019). Normal polysomnography parameters in healthy adults: A systematic review and meta-analysis. Lancet Respir Med..

[34] Figorilli M (2017). Comparison between automatic and visual scorings of REM sleep without atonia for the diagnosis of REM sleep behavior disorder in Parkinson disease. Sleep.

[35] Kim T, Kim JW, Lee K (2018). Detection of sleep disordered breathing severity using acoustic biomarker and machine learning techniques. BioMed. Eng. Online.

[36] Alshaer H, Hummel R, Mendelson M, Marshal T, Bradley TD (2019). Objective relationship between sleep apnea and frequency of snoring assessed by machine learning. J. Clin. Sleep Med..

[37] Kempfner J (2014). Rapid eye movement sleep behavior disorder as an outlier detection problem. J. Clin. Neurophysiol..

[38] Guttowski D, Mayer G, Oertel WH, Kesper K, Rosenberg T (2018). Validation of semiautomatic scoring of REM sleep without atonia in patients with RBD. Sleep Med..

