# Supplementary material for: RBDtector: an open-source software to detect REM sleep without atonia according to visual scoring criteria
Source: Sci Rep. 2022 Dec 3;12:20886. doi: 10.1038/s41598-022-25163-9 (PMC9719467; doi:10.1038/s41598-022-25163-9)
Supplement: Supplementary file 1 — Supplementary Information. [file 41598_2022_25163_MOESM1_ESM.zip › Supplementary Material.docx]

**Supplementary Material:**

**Table s1: Performance of *RBDtector* using the original cut-off values published by the SINBAR group.**^1^

| **RSWA metric** | **Published cuf-off**^1^ | **Sensitivity** | **Specificity** | **Accuracy** |
| --- | --- | --- | --- | --- |
| **Mentalis, phasic** | **16.3%** | **74%** | **98%** | **84%** |
| **Mentalis, tonic** | **9.6%** | **42%** | **100%** | **65%** |
| **Mentalis, any** | **18.2%** | **78%** | **98%** | **86%** |
| **FDS, phasic** | **16.8%** | **92%** | **95%** | **94%** |
| **SINBAR (Mentalis, any + FDS, phasic)** | **31.9%** | **86%** | **100%** | **91%** |

Abbreviations: FDS: flexor digitorum superficialis; RSWA: REM sleep without atonia


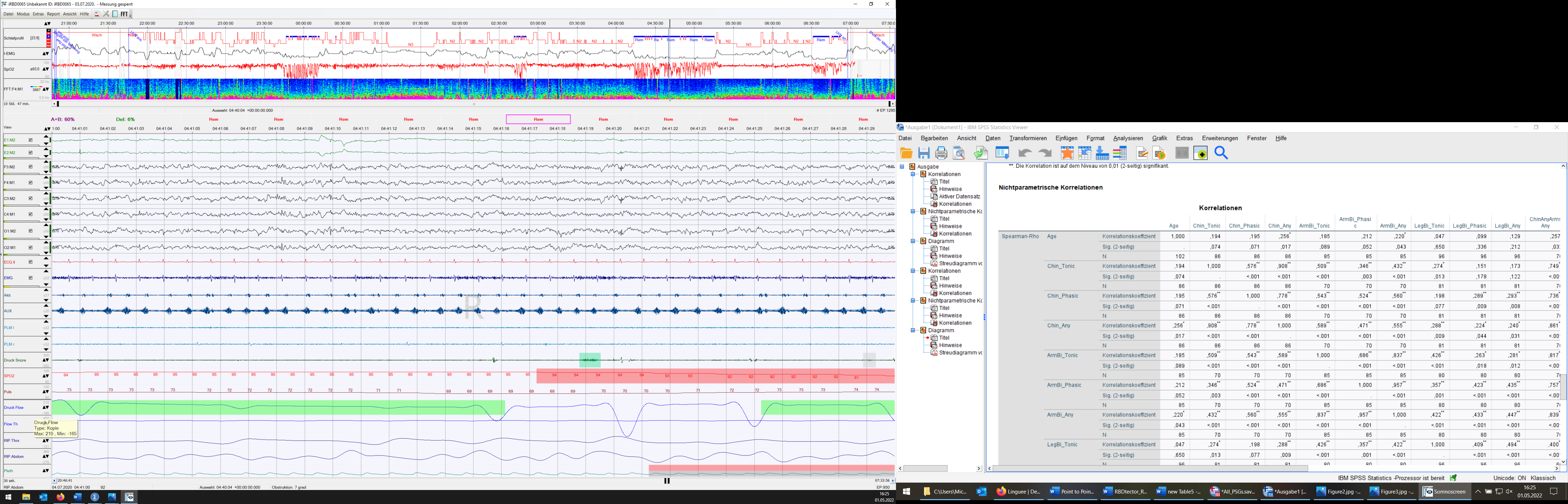


EOG

F3:M2

F4:M1

C3:M2

C4:M1

O1:M2

O2:M1

EKG

Mentalis

FDS R

FDS L

TA R

TA L

**Figure s1: Example of technical artifact on both FDS EMG channels.** Both FDS EMG channels exhibited an almost oscillating waxing- and waning artifact during the whole night without correlation to EKG or breathing


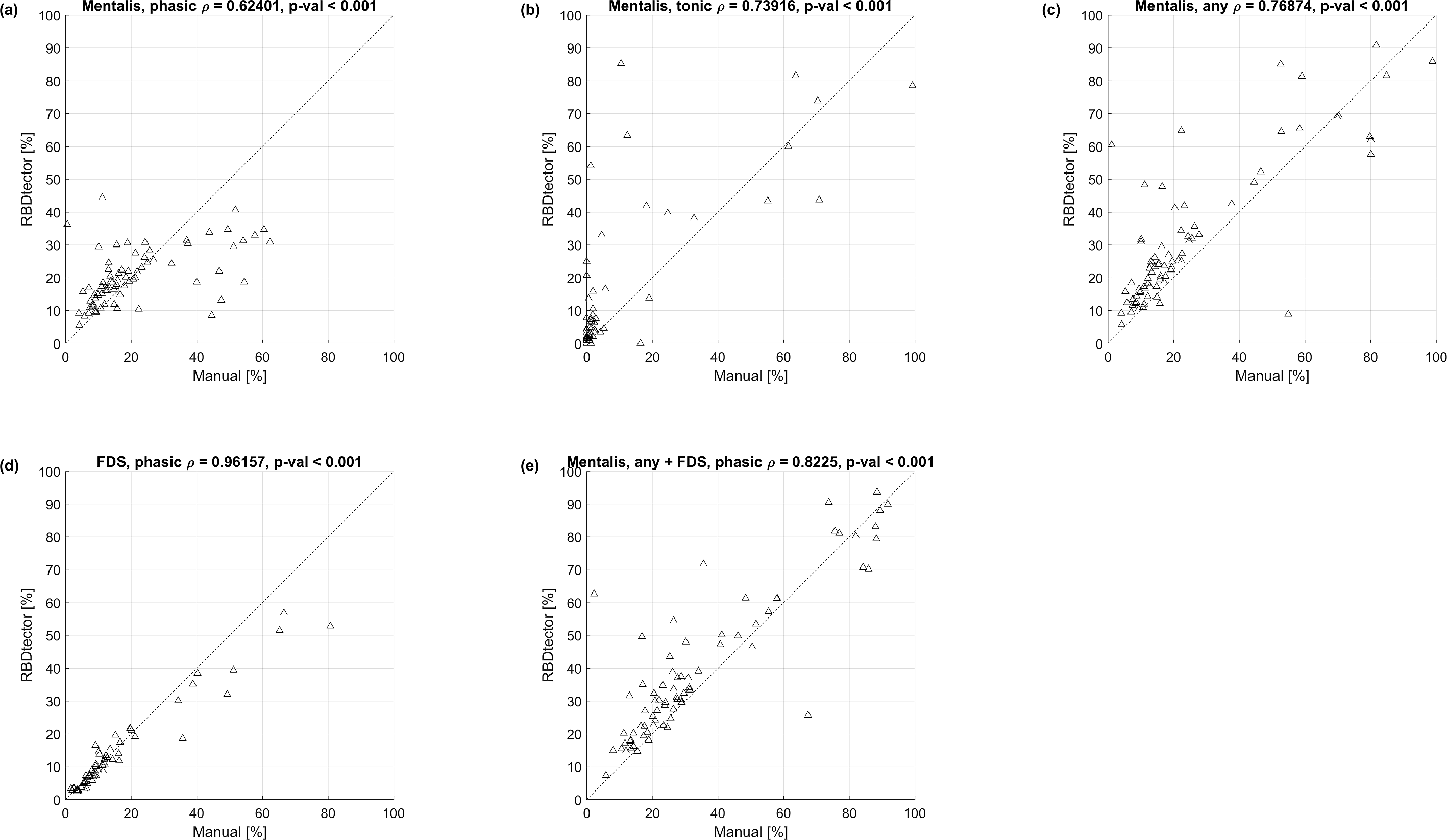


**Figure s2**: **Correlation plots of the *RBDtector* scorings compared to the manual scoring performed on a previoulsy published dataset**.^2^ The scorings shown are: (a) mentalis phasic, (b) mentalis tonic, (c) mentalis any, (d) FDS bilateral phasic, and (e) mentalis any + FDS bilateral phasic. Line of unity are dashed. For each correlation, the Spearman ρ value and its p-value are shown. Abbreviations: FDS, flexor digitorum superficialis muscle.

**Table s2: Comparison of performance detection for RBD achieved in a previously published dataset.**^2^ The data obtained from the publication of Frauscher et al.^2^ are shown as mean and 95% confidence interval.

| Index | Performance | Manual^2^ | Automatic without artifact correction^2^ | *RBDtector –*removal of arousals only |
| --- | --- | --- | --- | --- |
| Mentalis, phasic | Sensitivity | 0.90 (0.68-0.99) | 0.90 (0.68-0.99) | 0.80 |
|  | Specificity | 0.68 (0.55-0.79) | 0.47 (0.33-0.60) | 0.40 |
| Mentalis, tonic | Sensitivity | 0.64 (0.38-0.82) | 0.75 (0.51-0.91) | 0.80 |
|  | Specificity | 1.00 (0.92-1.00) | 0.98 (0.91-1.00) | 0.93 |
| Mentalis, any | Sensitivity | 0.85 (0.62-0.97) | 0.90 (0.68-0.99) | 0.85 |
|  | Specificity | 0.73 (0.60-0.84) | 0.45 (0.32-0.58) | 0.39 |
| FDS, phasic | Sensitivity | 0.94 (0.73-1.00) | 0.95 (0.72-1.00) | 0.94 |
|  | Specificity | 0.87 (0.75-0.94) | 0.81 (0.69-0.90) | 0.82 |
| Mentalis, any + FDS, phasic | Sensitivity | 0.83 (0.59-0.96) | 0.94 (0.73-1.00) | 0.83 |
|  | Specificity | 0.87 (0.75-0.94) | 0.72 (0.59-0.83) | 0.60 |

**Reference**

1. Frauscher, B. *et al.* Normative EMG Values during REM Sleep for the Diagnosis of REM Sleep Behavior Disorder. *Sleep* **35**, 835–847 (2012).

2. Frauscher, B. *et al.* Validation of an integrated software for the detection of rapid eye movement sleep behavior disorder. *Sleep* **37**, 1663–1671 (2014).
